# Two-Layer Inkjet-Printed Microwave Split-Ring Resonators for Detecting Analyte Binding to the Gold Surface

**DOI:** 10.3390/s24051688

**Published:** 2024-03-06

**Authors:** Matthias Paul, Harald Kühnel, Rudolf Oberpertinger, Christoph Mehofer, Doris Pollhammer, Markus Wellenzohn

**Affiliations:** 1 Department of Engineering, Applied Electronics and Technical Informatics, University of Applied Sciences Vienna (FH Campus Wien), 1100 Vienna, Austria; matthias.paul@fh-campuswien.ac.at (M.P.); rudolf.oberpertinger@fh-campuswien.ac.at (R.O.); 2 Competence Center for IT-Security, Department of Engineering, University of Applied Sciences Vienna (FH Campus Wien), 1100 Vienna, Austria; 3 Department of Applied Life Sciences, Bioengineering, University of Applied Sciences Vienna (FH Campus Wien), 1100 Vienna, Austria; harald.kuehnel@fh-campuswien.ac.at (H.K.); poodor@yahoo.de (D.P.); 4 Department of Engineering, High Tech Manufacturing, University of Applied Sciences Vienna (FH Campus Wien), 1100 Vienna, Austria; christoph.mehofer@fh-campuswien.ac.at

**Keywords:** inkjet-printed sensor, two-layer microwave split-ring resonator, biofunctionalization, photonic sintering, biosensor, high-frequency technology

## Abstract

This work focuses on demonstrating the working principle of inkjet-printed Au nanoparticle (NP) two-layer Gigahertz (2.6 GHz) microwave split-ring resonators (SRRs) as a novel platform for the detection of analytes on flexible substrates. In contrast to the standard fabrication of split-ring resonator biosensors using printed circuit board technology, which results in a seven-layer system, the resonators in this work were fabricated using a two-layer system. A ground plane is embedded in the SRR measurement setup. In this method, a microwave electromagnetic wave is coupled into the Au SRR via an inkjet-printed Cu-NP stripline that is photonically sintered. This coupling mechanism facilitates the detection of analytes by inducing resonance shifts in the SRR. In this study, the functionality of the printed sensors was demonstrated using two different Au functionalization processes, firstly, with HS-PEG7500-COOH, and, secondly, with protein G with an N-terminal cysteine residue. The sensing capabilities of the printed structures are shown by the attachment of biomolecules to the SRR and the measurement of the resulting resonance shift. The experiments show a clear shift of the resonance frequency in the range of 20–30 MHz for both approaches. These results demonstrate the functionality of the simplified printed two-layer microwave split-ring resonator for use as a biosensor.

## 1. Introduction

As a non-contact printing method, inkjet printing offers several advantages over traditional manufacturing, such as eliminating the need for physical masks, reducing material waste, and allowing the precise control of material deposition, making it suitable for producing complex structures with a high resolution and accuracy. In addition, inkjet printing can be applied to a wide range of materials, including metals, polymers, ceramics, and biomaterials, making it a versatile platform for diverse applications. For example, inkjet printing has been used to produce electronic circuits, antennas, sensors, and solar cells [1,2,3,4]. Gold surfaces are widely used for biosensors [5,6] because they are very amenable to biofunctionalization and there is a wealth of experience and literature on the subject. The successful detection of analytes using printed SRRs holds great promise for the development of reliable and cost-effective biosensors for medical and environmental applications.

Overall, this work represents a promising step towards the development of practical, real-world applications for inkjet-printed Au-NP SRRs on a flexible Kapton substrate in analytical chemistry and biosensing.

Antibody-based bioassays are the main technology for biomolecule detection. Anti-bodies are highly specific to their antigen and bind with high affinity. Immunoassays measure the antibody–antigen interaction mainly immobilized on materials such as gold, glass, and plastic. The immobilization of the antibody on these surfaces is one of the essential steps in preparation for these assays. There are different ways to achieve the immobilization of antibodies on gold surfaces. Firstly, antibodies can physically absorb to the gold, which is the simplest way to immobilize the antibody. The antibodies are absorbed with a random orientation, which could lead to denaturation during this process, yielding poor reproducibility [7]. In contrast, covalent binding is more reproducible than random adsorption. Drawbacks include disordered antibody orientation which results in less biological activity [8]. A second approach is using protein G, which leads to an oriented binding of the antibody by binding to the Fc region. The thiol group of an additionally bound cysteine leads to properly oriented protein layers of protein G on the gold surface [9].

**Figure 1:**
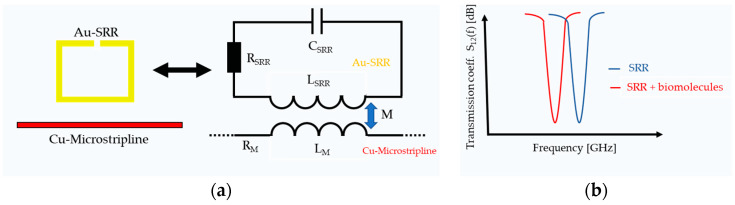
(**a**) Schematic diagram of a stripline and an SRR and the corresponding coupling mechanism of the stripline with the SRR, which physically forms an RLC resonant circuit with a specific resonant frequency; (**b**) the resonance in the transmission coefficient S_12_ of the system without (blue) and with (red) biomolecular coupling, where binding of molecules to the sensor surface leads to a resonance shift.

In several studies, sensors based on high-frequency technology have also been investigated for their suitability as biosensors. In this context, the idea of developing sensors for biomedical applications based on microwave split-ring resonators is also being pursued [10,11,12,13,14,15,16]. A microwave split-ring resonator typically consists of circular geometric structures with dimensions in the millimeter or centimeter range. The circular shapes and rectangular wire structures contain a gap. The metallic structure of the SRR is applied to a dielectric substrate such as FR1, FR4, Rogers-materials, etc., which has a metallic layer on the opposite side that is typically made of copper material and represents the ground potential. A microwave transverse electromagnetic (TEM) mode is generated and propagated along a printed microstrip line. The split-ring resonator is aligned parallel to the strip line in proximity. This allows the mode to couple into the metallic SRR. Physically, the microstrip line and the SRR split ring form an RLC resonant circuit [10,12,15]. Figure 1 shows the basic working principle of the SRR sensor system.

The resonant frequency of the SRR system can be determined by measuring the frequency-dependent scattering parameters Sij(f), such as the transmission coefficient S_12_(f) or the reflection coefficient S_11_(f), using a vector network analyzer. Using the simplified RLC circuit model, the material losses are represented by the Ohmic resistance, the gap of the resonator can be understood as a capacitance, and the nearly closed loop and the stripline lead to the inductance of the system. The SRR sensor system has specific electromagnetic resonance properties that depend on the geometry and physical properties of the material, as well as the respective layer thicknesses. The resonance frequency can be influenced by changing the dimensions or geometries of the structures or the material parameters. The gap region is the part of the sensor with the highest electric field strength [12]. This small area of the sensor surface is chemically functionalized so that only certain molecules can bind or attach to it. The binding of the biomolecules causes a shift in the resonance frequency that can be measured (Figure 1). Without this chemical functionalization, there would be no specific binding of molecules to the sensor surface and, therefore, no shift in the resonance frequency.

For the realization of biosensors with SRRs, the geometries of the resonators are chosen in the range of a few centimeters to millimeters. The operating range of these sensors is, therefore, typically in the frequency range of a few GHz to around 13 GHz. In contrast to SRR biosensors, plasmon-based biosensors typically use the visible frequency range, where biomolecules exhibit the characteristic absorption or scattering spectra. Plasmons are generated by collective oscillations of electrons in a metallic conducting material. Typically, plasmons are excited by electromagnetic waves at visible wavelengths and propagate at interfaces between a metal and a dielectric [17,18]. Therefore, these types of sensors have major differences compared to SRR-based biosensors. In summary, the main differences lie in the operating frequency range, the physical mechanism of excitation, and the localization of the electric fields.

In the microwave field, split-ring resonators are typically fabricated using printed circuit board technology. For biosensors, the sensor surfaces are made of a thin layer of gold, as this allows easy chemical functionalization and the high binding of molecules. The production of these sensors using printed circuit board technology results in a seven-layer system, as gold cannot be deposited directly onto a copper surface for stability reasons and, therefore, requires an intermediate layer of nickel. This leads to a structure comprising seven layers (Au/Ni/Cu/Substrate/Cu/Ni/Au), with respective thicknesses of, typically, 0.1 μm, 5 μm, 35 μm, 1.55 mm, 35 μm, 5 μm, and 0.1 μm. The substrates used are typically rigid materials such as FR1, FR4, or Rogers- materials. In contrast to the standard PCB fabrication, which results in a seven-layer system, the resonators in this work were fabricated using inkjet printing and photonic sintering, allowing the biosensor to be fabricated via a two-layer system. In the discussion (Section 4), a comparison of published works on conventionally manufactured SRR biosensors and the presented sensor from this work is provided.

**Figure 2:**
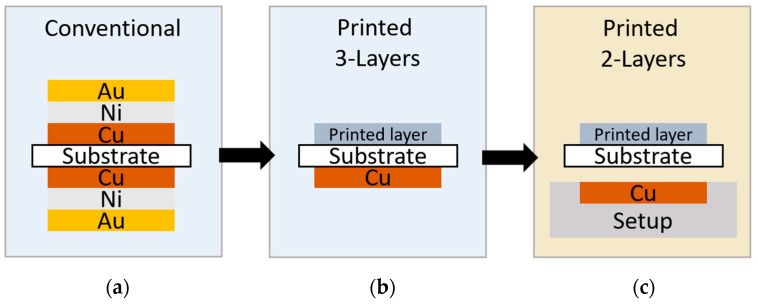
(**a**) Conventional 7-layer fabrication; (**b**) inkjet-printed 3-layer fabrication; and (**c**) printed 2-layer fabrication with the ground potential in the measurement setup.

A sensor operating in the microwave range requires a ground potential on the rear side, e.g., based on a copper layer, and would, therefore, must be implemented as a three-layer system (Figure 2). In this work, the ground potential is already considered in the measurement setup, which makes it possible to produce the sensor using a two-layer system. The inkjet printing of sensors is a very simple method of manufacturing, and the addition of photonic sintering allows production in a roll-to-roll process. This can save a lot of material, time, and cost during production.

In this paper, we show that SRR sensors based on the two-layer system have comparable functionality to SRR sensors fabricated from a seven-layer system using printed circuit board technology. The basic functional principle of inkjet-printed SRRs was already demonstrated by the authors in a two-page conference paper [19].

## 2. Materials and Methods

**Figure 3:**
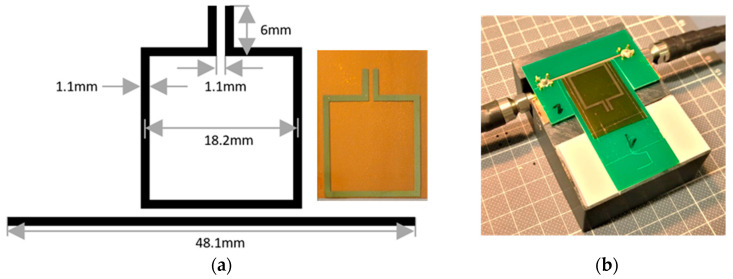
(**a**) Geometry of the 2.6 GHz Au SRR with increased measurement slit, the Cu stripline and the printed Au SRR on Kapton foil; and (**b**) SRR insertion setup with 3D-printed filament for positioning.

The printing process is performed with a Materials Inkjet Printer from Dimatix (DMP-2850) equipped with 10-pl-nozzled cartridges [20]. The gold layer of the SRRs is manufactured by printing 3–5 nm particles in an ink solution from UTDOT (UTDAu25IJ), which is optimized for inkjet printing in terms of viscosity and contact angle, matching the printer nozzles and substrate. Additionally, the parameters of the printer must be adjusted to the specifics of the ink. For the present gold ink, this results in a surface temperature of 30 °C, a printhead temperature of 25 °C, a max. jetting frequency of 5 kHz, a jetting voltage of 25 V ± 1 V depending on individual nozzle performance, a resolution of 1270 dpi, and a total of two printed layers. A 50 µm-thick flexible Kapton material (200 HN from DuPont, Luxembourg) is used as a substrate. The resonance frequency of the printed Au SRR depends strongly on the material thickness and the frequency-dependent susceptibility of the ink. Here, the geometry and printing conditions of the rectangle resonator with a symmetric slit and increased measurement region are designed for a resonant frequency of 2.6 GHz. The geometry and measurement setup are shown Figure 3 and are also available in a published preliminary study by the group [2].

The coupling stripline is printed using ink containing 110–130 nm copper particles from Novacentrix (Metalon ICI-002HV, Austin, TX, USA). The straight line is printed on the solid FR1 platform. The geometry and thickness (adjusted by the number of layers) of the stripline are designed to achieve 50 Ω lossless coupling. For the present copper ink, this results in a surface temperature of 30 °C, a printhead temperature of 25 °C, a max. jetting frequency of 5 kHz, a jetting voltage of 35 ± 1 V depending on individual nozzle performance, a resolution of 1693 dpi, and a total of two printed layers. The presented values can differ depending on the shelf life of the inks. To avoid agglomeration of the nanoparticles and subsequent clogging of the printer nozzles, the inks are filtered before they are filled into the cartridges.

**Figure 4:**
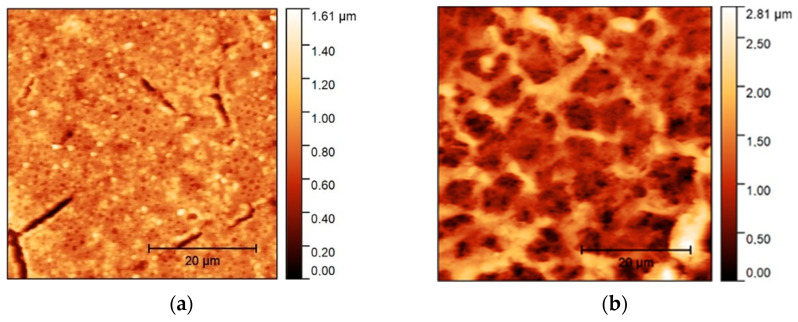
Atomic force microscope recordings of printed Au nanoparticles on Kapton: (**a**) nanoparticles on surface before sintering; and (**b**) connected sintering network surface after sintering.

After printing, the nanoparticles are covered loosely on the substrate surface. To achieve a solid and electrically conductive structure, one must apply energy to melt the nanoparticles together. This can be achieved via several approaches including selective laser sintering [21], intense pulsed light sintering [22], or thermal sintering [23]. The Cu stripline was sintered by phonically flashing the material with a high-power pulse from a xenon flash lamp. The discharge (two capacitors with 4700 µF at 480 V) takes place in a few milliseconds and results in a homogeneous material with a thickness of 2.19 ± 1.03 µm (after 13 measurements with the 2D profilometer Surtronic S128, AMTEK Taylor Hobson Weiterstadt, Germany) and a conductivity of 2.54 ± 0.82 Ω/□. The pulse energy must be adjusted for every object according to the material (melting temperature), thickness, and substrate (reflection). A special mirror head was designed by the group to achieve a homogeneous distribution of the flash pulse, and homogeneous sintering and conductivity. Figure 4a shows an atomic force microscope (Nanosurf NaioAFM, Liestal, Switzerland) recording of the unsintered gold nanoparticles, spread over the substrate surface. The applied photonic energy leads to the formation of a conductive sintering network, as shown in Figure 4b. The energy transfer via photons deposits the energy very specifically on the metallic nanoparticles, heating them to their specific melting point, which strongly depends on particle size, e.g., for gold [24] and copper [25]. By choosing the size of the nanoparticles, the melting temperature can be lowered, allowing them to be used with temperature-sensitive substrates. In addition, the short photon pulse, which lasts only a few milliseconds, is gentle on the substrate due to the short energy impact. Another sintering approach more suitable for structures printed on substrates with higher melting points would be thermal sintering. For the printed Au SRR, heating to 250 °C for 20 min results in a conductivity of 0.98 ± 0.3 Ω/□.

The coupling of the microwave signals from the R&S ZNB20 network analyzer into the printed structure is carried out by SMA connectors. They are glued to the Cu stripline using Ag adhesive paste. A FLM 3D-printed platform holds the assembly in place and provides a ground potential plane for the SRR. The ground plane is represented by copper foil glued to the platform, spreading below the region of the SRR while being connected to the ground potential of the network analyzer.

To introduce biosensing capabilities, the Au surface is treated in several steps to achieve optimal attachment of the analyte. For biofunctionalization, a 6 mm radius area is confined by a rubber mask on the slit, where the electric field, and, thus, the influence on the frequency shift, is the largest [12].

The evaluation and the configuration of the biofunctionalization was performed by an ELISA-like method, where the antibody was not immobilized by hydrophobic interactions to the polymer surface but was bound to gold by two different methods, HS-PEG7500-COOH with ammine coupling and S-protein G.

Round areas of gold that corresponded to the size and shape of a 96- or 12-well plate were printed on 50 µm-thick flexible Kapton material (200 HN from DuPont), and then punched out via a perforator. The clean platelets (30 min ultrasound bath in 99.9% ethanol) were placed in the wells facing the gold side up. HS-PEG (HS-PEG7500-COOH JKA5101-1G Merck KGaA, Darmstadt, Germany) was, then, bound to the gold surface (60 min in EtOH). Antibodies were bound by activation via amine coupling (15 min NHS-EDC), capture antibody (30 min, Anti-Human IgG (Fc specific) antibody produced in goat (I2136 Merck KGaA, Darmstadt, Germany), deactivation of additional reactive groups (7 min, Ethanolamine-HCl), and blocking of additional groups with bovine serum albumin (60 min, 2% BSA in PBST). Attachment of the analyte antibody (60 min, adalimumab human-IgG) was performed thereafter.

The binding of adalimumab [26] to the surface was detected with an anti-human polyclonal antibody labeled with an alkaline phosphatase (detection antibody (Goat anti-Human IgG, Fc, AP-conjugate, Millipore AP113A, Merck KGaA, Darmstadt, Germany)). This binding was evaluated by measuring the absorbance of a colorimetric reaction product at 405 nm, which is caused by an enzyme substrate (pNpp P4744, Merck KGaA, Darmstadt, Germany). The signal was proportional to the analyte binding. This arrangement was used to investigate whether the setup of the assay works in principle, as well as for setting up the whole biofunctionalization procedure. Furthermore, we tested whether a non-specific binding to the gold and/or non-specific binding of the antibodies used took place and determined the optimal regeneration conditions. Gold functionalization with S-protein G was tested the same way.

In this study, the functionality of the printed sensors was demonstrated using two different methods. The first functionalization of the gold surface was performed with HS-PEG7500-COOH (P1). The functionalization steps include: cleaning of the Au surface (30 min ultrasound bath in 99.9% Ethanol), Au–thiol coupling and pegylation (60 min with HS-PEG7500-COOH in EtOH, Cytiva Amine Coupling Kit, Cytiva, Marlborough, MA, USA), activation via amine coupling (15 min NHS-EDC), attachment of capture antibody (30 min, anti-human-IgG), deactivation of additional reactive groups (7 min, Ethanolamine-HCl), blocking of additional groups with bovine serum albumin (60 min, 2% BSA in PBST), and, finally, attachment of the analyte (60 min, adalimumab human-IgG). The second method for functionalizing the gold surface was achieved using protein G (NBP2-34962, Novus Biologicals, Littelton, CO, USA) with an N-terminal cysteine residue (S-protein G) (P2). The gold surfaces were cleaned by sonification in absolute ethanol. Then, one drop (50–100 µL) of S-protein G (200 µg/mL in PBS) was applied to gold surface for 20 min. For a blocking step, to prevent unspecific binding, one drop (appr. 50–100 µL) of 2% BSA in PBS was applied and incubated for 20 min. One drop (appr. 100 µL) of Humira IgG (128 µg/mL in PBS) was applied and incubated for 30 min [9]. Between every step, the surface was cleaned with PBS, and a measurement was performed.

To demonstrate the functionality of the functionalized sensors, the SRR sensors were precisely positioned relative to the stripline using an insertion system. SMA connectors were attached to the printed stripline using conductive Ag adhesive paste and were connected to the vector network analyzer (ZNB20, Rohde&Schwarz, Munich, Germany). In contrast to the standard fabrication of split-ring resonators using printed circuit board technology, where biosensors are typically constructed from a seven-layer system, the resonators in this work were fabricated using a two-layer system, where the SRR insertion setup includes a ground plane on which the sensor is positioned. The scattering matrix elements S_ij_(f) are measured as a function of frequency. In this study, the transmission coefficient S_12_(f) was used to determine the resonant frequency of the resonator. The binding of the biomolecules to the functionalized gold surfaces results in a shift of the resonance frequency, which is measured.

**Figure 5:**
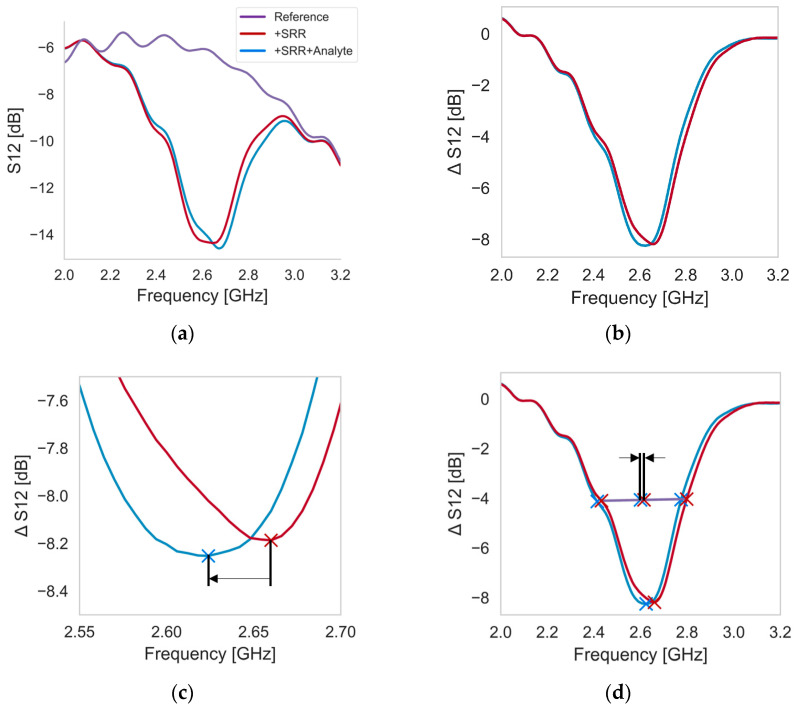
Example of a measurement of one SRR biofunctionalized with Process 2; (**a**) raw signal of the reference curve, with SRR and with SRR including biofunctionalization in S_12_ (dB); (**b**) calculated resonance signal (S_12_-signal minus S_12_-reference); (**c**) minimum shift of the resonance frequency; and (**d**) FWHM shift of the resonance curves.

The signal transmission through the stripline (S_12_) determines the resonance for each processing step (Figure 5a). To prevent signal interference through the measurement setup, a reference measurement is performed without an SRR for each experiment. All measured signals are then taken in reference to this reference curve to obtain the true resonance (Figure 5b). Because of possible ripples in the signal, additional mathematical methods are introduced to obtain a broader picture of the shift in resonance frequency.

To increase the number of data points in the region around the minimum, a regression function is calculated for a small region around the minimum, and the shifts are calculated with respect to these curves (Figure 5c). Secondly, the full width at half maximum (FWHM) is calculated to also account for the curvature of the resonance signal. Therefore, the frequencies (f) matching half of the minimum value (dB) are determined, and the mean of these numbers represents the resonance frequency (Figure 5d).

## 3. Results

**Figure 6:**
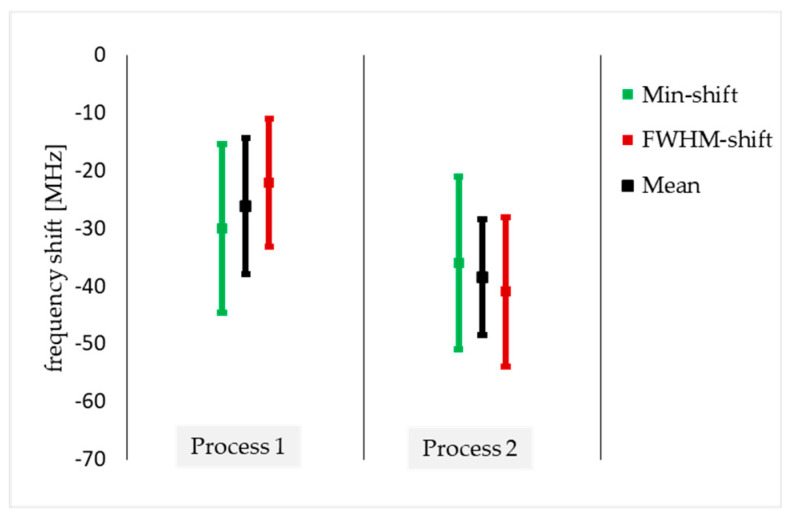
Total S_12_ resonance frequency shift in MHz for both biofunctionalization processes and both evaluation methods combined from two experiments on three individual SRRs each. The mean value results from all evaluation methods, depending on the process used.

The resulting shifts in resonance frequency for both biofunctionalization processes is shown in Figure 6. The corresponding values are listed in Table 1, showing a clear shift in resonance frequency for both processes and both evaluation methods. In addition to the frequency shift, one can compute the corresponding sensitivity S based on the concentrations employed in the respective biofunctionalization processes from above (Table 1). To calculate the sensitivity, the frequency shift of the process is divided by the concentration of the analyte. In both processes, the concentration was 128 µg/mL. For the 100 µL analyte, this results in 2.04 MHz/µg (P1) and 3.01 MHz/µg (P2). The Q-factor can be determined for the resultant curves by dividing the resonance frequency by the frequency span at −3 dB [12]. Calculating the Q-factor of two experiments in P2 results in 9.8 ± 0.16. The minor variations in the Q-factor were anticipated given the slight changes in the curves as they can be observed, e.g., in Figure 5.

The standard deviations in frequency and sensitivity are too large to differentiate clearly between the two processes, but Process 2 exhibits a slightly larger shift. The resonant frequency strongly depends on the positioning of the SRR near the stripline. We determined the deviation of the resonance frequency from inserting the SRR into the measurement setup. This resulted in a resonance shift of 1–6 MHz (minimum) and 3–10 MHz (FWHM) inert to the insertion positioning in the measurement setup. Further, the positioning of the droplet on the measurement slit and the droplet volume in the biofunctionalization process slightly varies from experiment to experiment. Considering the above, further modifications must be applied to the measurement setup to decrease the standard deviation and to be able to detect more subtle changes in the material attached to the gold surface. This could include improving the measurement platform and introducing microfluidic setups.

Additional contact angle measurements on functionalized gold surfaces showed a distinct decrease in the contact angle when HS-PEG7500-COOH (like used in Process 1) was incubated and washed with PBS (Table 2). This suggests a stable formation of an HS-PEG7500-COOH SAM layer on the inkjet-printed Au [27].

**Figure 7:**
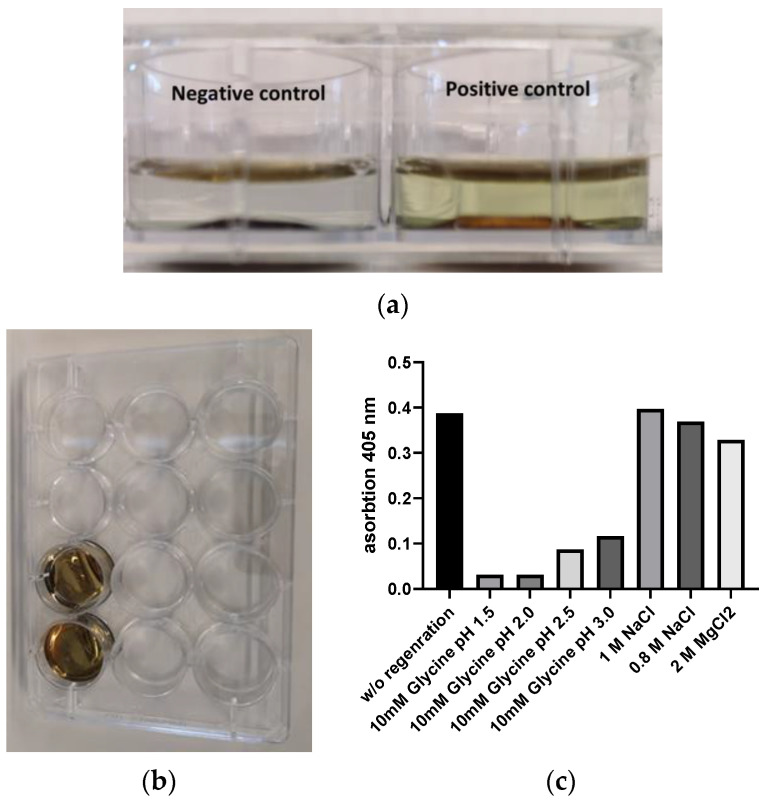
(**a**) ELISA-like setup for evaluation of biofunctionalization of gold platelets on the left side (negative control (no antibody was immobilized)) without staining of the substrate on the right side with yellow staining due to antibody immobilization and detection by anti-human antibody labeled with alkaline phosphatase; (**b**) image of a 12-well plate with gold platelets representing the configuration used, and 96-well plates were prepared the same way; and (**c**) regeneration study with different regeneration solutions; an intense pH-dependent decrease in signal induced by pH switch can be observed; and high salt regeneration solutions did not affect antibody binding to a large extent.

The binding of the antibody to the gold surface with a linker of HS-PEG7500-COOH was confirmed by experiments using an ELISA-like setup (Figure 7a). Different solvents and incubation times for HS-PEG7500-COOH immobilization were tested; ethanol was the one with the best properties. Further studies, like regeneration solution determination, were carried out. The removal of adalimumab from the covalently bound polyclonal anti-human antibody was tested. In addition, 10 mM Glycine solutions with pH values of 1.5, 2.0, 2.5, and 3.0 were tested. The treatment showed a clear decrease in bound adalimumab with decreasing pH. On the contrary, salt (NaCl) concentrations of 1.0 and 0.8 M showed nearly no regeneration efficiency, while 2 M MgCl2 did show low regeneration (Figure 7c). Finally, a regeneration solution of pH 2.3 was used to prevent damage to the covalently bound antibody while maintaining efficient regeneration conditions.

Additionally, we assessed the non-specific binding of the detection antibody to the gold surface and the antibody bound to HS-PEG7500-COOH. Observably, the binding of the antibody occurred without amine coupling to the gold surface. The split-ring experiments were conducted under optimal conditions as detailed in the Materials and Methods section. In the case of the S-protein G setup, the arrangement was also pretested using an ELISA-like setup, and optimal conditions were subsequently employed for split-ring functionalization, as outlined in the Section 2.

## 4. Discussion

Of the two setups tested, HS-PEG7500-COOH and S-protein G, S-protein G shows clear advantages compared to the HS-PEG7500-COOH method. The S-protein G method is simpler and uses fewer chemicals, which are partly poisonous. The experiments with an ELISA-like setup confirmed the binding and provided a deeper understanding of the assay. Introducing a reusable sensor would be advantageous for the further use of this setup for the detection of human antibodies. Moreover, multiple loadings of the sensor would be advantageous for the future evaluation of the binding constants. In this context, regeneration studies showed that regeneration is possible. The immobilization of the antibody with S-protein G is very simple and binding can also be covalently crosslinked. With the successful validation of the immobilization approach, opportunities for enhancement emerge. We could use an optimized anti-human capturing antibody with a matched regeneration solution. Furthermore, the detection of larger particles like extracellular vesicles becomes feasible.

The printer used in this work could handle various inks containing nanoparticles. The particle size is limited by several factors. Firstly, the particles must be small enough to pass through the printer nozzles (10 pl per drop). To avoid clogging, additional filtering must be performed (~200 nm filter size). This limits the particle size to about 150 nm. Metallic nanoparticles, especially, need sintering after the printing process. Depending on the sintering process (intense pulsed light (ILP), selective laser sintering, or thermal sintering), a large particle size could lead to the need for large energies or temperatures, which could exceed the limitations of the substrate. Furthermore, the nanoparticles must be in a homogeneous dispersion during the printing process to avoid deposits at the bottom of the vessel, resulting in clogging. Due to these effects, the inks are typically only usable for short periods of time. Another important aspect is the material pairing between the ink and substrate. Not every nanoparticle ink can be combined with every substrate material. The surface energy of the solid and the surface tension of the ink are decisive factors in this regard. This also plays an essential role in multilayer printing.

Inherent to the inkjet printing method, one cannot print perfectly shaped lines or borders. Placing single dots next to each other always results in a line waviness that is dependent on the drop distance (dpi) and the spreading of the ink on the substrate.

An excerpt of conventionally manufactured SRRs by using PCB technology is provided in Table 3. There are three papers that especially match the gold–biomolecules attachment processes from the approaches above. Lee et al. [11] labeled a seven-layer SRR (11.5 GHz) with protein G and used an antibody-PSA interaction to measure the biomolecules. Depending on the PSA concentration varying from 1 to 10 ng/mL, they measure a shift of −5 to −20 MHz. The same group also published an improved sensor design [28] with an asymmetric SRR (11.25 GHz). They used the same Au attachment method via protein G but measured cortisol concentration from 1 to 300 ng/mL, resulting in a shift of −3.85 to −76.92 MHz. Considering that the sensitivity should increase with the resonance frequency, the presented numbers from the present paper are highly compatible. Jaruwongrungsee et al. [29] also used standard fabrication processes to manufacture a 1.8 GHz SRR. They measured the shift depending on IgG concentration (0–200 µg/mL) in DI water attached by thiol (SH) and amine coupling (NH2) via a microfluidic system on the Au surface. This resulted in a frequency shift of 6.5–13 MHz. For a 100 µg/mL IgG concentration (comparable to the 126 µg/mL used in this work), they measured a 9 MHz shift that calculates to a sensitivity of 0.09 MHz/(µg/mL).

With further modifications to the SRR design, like multiple resonators [11], thinning of the stripline, or asymmetric resonators [29,30], we should be able to improve the Q-factor and increase the sensitivity of the inkjet-printed SRR further, and, thus, decrease the relatively large standard deviation of the present results.

## 5. Conclusions

The inkjet-printed resonators, featuring a simplified two-layer setup, exhibited a resonance frequency of 2.6 GHz. Two biofunctionalization processes were introduced to determine the sensitivity of the sensor. The first approach uses Au–thiol coupling and pegylation (P1) and the second approach uses S-protein G (P2) for biomolecule (IgG) attachment on the printed Au surfaces. The study revealed a frequency shift ranging from −26.12 ± 11.07 MHz (P1) to −38.50 ± 12.95 MHz (P2) and a corresponding sensitivity of −2.04 ± 0.87 MHz/µg (P1) to −3.01 ± 1.01 MHz/µg (P2) depending on the biofunctionalization process. While the variation between measurements is too large to differentiate between functionalization processes, the shifts show a clear detection of biomolecules with the printed sensors. Comparing with the literature (Table 3) shows that SRR sensors based on the two-layer system perform comparably to those fabricated using PCB technology with a seven-layer system. Consequently, the experiments illustrated the operational principle and efficacy of the printed two-layer sensors. Given that printing and photonic sintering technologies are already utilized in industry in a roll-to-roll basis, there is potential to manufacture printed sensors for biomedical applications in large quantities at minimal expense. In a subsequent study, the SRR sensors will undergo optimization, and their functionality will be demonstrated under laboratory conditions with a developed liquid microfluidic chamber, using blood samples, for instance, to detect cytokines.

## Figures and Tables

**Table 1:** Resulting values for Process 1 (P1) and Process 2 (P2) from two experiments and three individual SRRs each.

	Minimum-Shift	FWHM-Shift	Mean Shift	Mean Sensitivity
P1	−30.06 MHz	−22.17 MHz	−26.12 ± 11.07 MHz	−0.204 ± 0.086 MHz/(µg/mL)
P2	−36.00 MHz	−41.00 MHz	−38.50 ± 12.95 MHz	−0.301 ± 0.101 MHz/(µg/mL)

**Table 2:** Resulting values for the contact angle change of water on inkjet-printed Au and additional incubation of HS-PEG7500-COOH after 41 measurements.

	Mean Contact Angle	Standard Deviation
Gold	79.05°	±2.75°
Gold + HS-PEG7500-COOH	37.41°	±4.04°

**Table 3:** Literature on pre-existing works on split-ring resonators from earliest to most recent.

Author	Analyte	Resonator	Manufacturing	Base Freq.	Shift
[11]	ss-DNA + Biotin -Streptavidin	4× double SRR	Galvanic etching	10.82 GHz	−160 MHz
[30]	ss-DNA	Round double SRR	Galvanic etching	12.35 GHz	−20 MHz
[31]	S-protein G + antibody-PSA 1–100 ng/mL	SRR	Galvanic etching	11.47 GHz	−5 to −20 MHz
[12]	Ethanol conc. in water	SRR	Galvanic etching	2.09 GHz	−130 MHz
[28]	S-protein G + cortisol 1–300 ng/mL	Asymmetric SRR	Galvanic etching	11.25 GHz	−3.8 to −76.92 MHz
[32]	Ethanol conc.	Double SRR	Galvanic etching	4.48 GHz	−250 MHz
[33]	FGF-2—Heparin 10–69 µg/mL	Round SRR	Galvanic etching	2.12 GHz	−240 to −420 MHz
[29]	SH-NH2 + Anti-IgG 50–200 µg/mL	SRR	Galvanic etching	1.79 GHz	−6.5 to −13 MHz
[34]	Ethanol conc. in DI water	Round resonator	Inkjet-printed	2.84 GHz	−90 MHz
This work	P1: SH-PEG + Anti-IgG 100 µg/mL	SRR	Inkjet-printed	2.65 GHz	−26.1 MHz
	P2: S-protein G + Anti-IgG 100 µg/mL	SRR	Inkjet-printed	2.65 GHz	−38.5 MHz

## Data Availability

The data presented in this study are available on request from the corresponding author.
